# Correlation between Quantitative ^13^C-Urea Breath Test and *Helicobacter pylori* Treatment Success in a Population-Based Cohort

**DOI:** 10.1155/2018/5439539

**Published:** 2018-11-13

**Authors:** Doron Boltin, Zohar Levi, Tsachi Tsadok Perets, Hemda Schmilovitz-Weiss, Rachel Gingold-Belfer, Ram Dickman, Iris Dotan

**Affiliations:** ^1^Division of Gastroenterology, Rabin Medical Center, Sackler Faculty of Medicine, Tel Aviv University, Israel; ^2^Gastroenterology Laboratory, Rabin Medical Center, Sackler Faculty of Medicine, Tel Aviv University, Israel

## Abstract

**Background:**

There are continual efforts to identify factors which influence the success of first-line therapy for *Helicobacter pylori* (*H. pylori*) infection. The ^13^C-urea breath test result (C13-UBT) utilizes *H. pylori* urease activity and is a highly accurate diagnostic assay. We aimed to determine whether the magnitude of C13-UBT result is related to treatment success.

**Methods:**

Adult patients who underwent a first-time ^13^C-urea breath test between January 2010 and January 2016 were included. In order to isolate a naïve test-and-treat population who were unlikely to have undergone an initial endoscopy-based *H. pylori* test, we excluded patients > 45 years and those with a previous C13-UBT. Data were extracted from the Clalit Health Services laboratory database.

**Results:**

A total of 94,590 subjects (36.1% male, age 28.5 ± 6.0 years) who underwent a first-time C13-UBT during the study period were included. C13-UBT was positive in 48,509 (51.3%) subjects. A confirmatory posttreatment C13-UBT was performed in 18,375 (37.8%), and eradication was successful in 12,018 (65.4%). The mean C13-UBT recording was 20.6 ± 16.2 DOB in subjects with successful eradication and 19.5 ± 13.1 DOB in subjects with treatment failure (OR, 1.01; 95% CI 1.00-1.01, *p* < 0.01). Among patients in the upper quintile of C13-UBT measurement, eradication was achieved in 67.6%, compared to 62.6% in the lower quintile (OR, 1.22; 95% CI 1.11-1.35, *p* < 0.01). Subjects in the top 1 percentile (C13-UBT ≥ 70 DOB) achieved eradication in 75.0%, compared to 65.3% among subjects with C13-UBT < 70 DOB (OR, 1.59; 95% CI 1.05-2.41, *p* < 0.01).

**Conclusions:**

The superiority in *H. pylori* eradication observed in subjects with a higher C13-UBT DOB is small but significant. Further studies should examine the physiological and microbiological basis for this finding.

## 1. Introduction

*Helicobacter pylori* (*H. pylori*) infection affects up to 50% of the world's population and is the leading cause of peptic ulcer disease, gastric cancer, and MALT lymphoma [1]. The most widely available and accurate noninvasive test for the diagnosis of *H. pylori* is the ^13^C-urea breath test (C13-UBT) [2]. This test exploits the urease-producing characteristic of the organism and involves measuring expired ^13^CO_2_ following oral ingestion of ^13^C-urea. When *H. pylori* infection is present, ^13^C-urea is cleaved by bacterial urease to 2NH_3_ and ^13^CO_2_. Absorbed ^13^CO_2_ is excreted via the lungs and measured in the expired air at baseline and 30 minutes following ingestion of the tracer. A positive result is indicated by an increase in expired ^13^C over baseline or delta over baseline (DOB) [3–5].

The magnitude of a DOB value is affected by multiple patient-related factors including gender, nationality, fasting, medications, and posture [6–9]. Test characteristics such as ^13^C-urea dosage, test meal, and test duration are also directly related to C13-UBT accuracy and may affect DOB magnitude [10–15].

DOB magnitude is also related to the severity of *H. pylori* infection. The density of *H. pylori* as well as histopathological chronicity and activity indices has been shown to positively correlate with DOB magnitude [16–25]. On the other hand, there appears to be no apparent correlation between DOB magnitude and dyspepsia or peptic ulceration [21, 25, 26].

Eradication of *H. pylori* remains a challenge, and there are continual efforts to identify factors which influence treatment success. The relationship between the histopathological severity of gastritis and the likelihood of treatment success is complex. Several studies have suggested that a higher degree of chronic active inflammation in the gastric mucosa is associated with *H. pylori* treatment success [27–30]. On the other hand, a high degree of mucosal atrophy decreases the likelihood of treatment success [30]. It is unclear whether the magnitude of C13-UBT result is related to treatment success. The aim of this study was to examine whether the C13-UBT result is related to the likelihood of subsequent successful eradication of *H. pylori*.

## 2. Materials and Methods

### 2.1. Patients

We retrospectively identified adult patients > 18 years who underwent a first-time ^13^C-urea breath test (C13-UBT) between 1 January 2010 and 31 December 2015 in Clalit Health Services (CHS). In order to exclude patients who may have previously received antibiotic treatment for *H. pylori*, we first excluded patients ≥ 45 years old who may have undergone an initial endoscopy-based *H. pylori* test in accordance with guidelines at the time of inclusion [31]. Subsequently, we excluded subjects who had undergone a C13-UBT in the 2 years prior to inclusion (1 January 2008-31 December 2009). Finally, we excluded patients who underwent a C13-UBT following a negative test. For the purpose of this study, we assumed that antibiotic treatment was administered following a positive C13-UBT and prior to a subsequent C13-UBT.

### 2.2. Sample Acquisition and Analysis

Breath samples were obtained from CHS facilities which incorporate 14 hospitals and 1300 primary care and referral clinics throughout eight districts. Breath tests were conducted by dedicated nurses. Patients were given 75 mg of ^13^C-labeled urea mixed with a test meal of 100 ml orange juice. All C13-UBT breath samples were transported and processed at a central laboratory at Rabin Medical Center, Petah Tikva, Israel. Samples were analyzed with a Gilson XL222 Automatic Breath Sampler (Gilson, Middleton, WI, USA) and an AP2003 Isotope Ratio Mass Spectrometer (IRMS) (Analytical Precision, Phoenix, AZ, USA). The ratio of expired ^13^C and ^12^C measured in parts per thousand was obtained at baseline and 30 minutes following ingestion of ^13^C-urea (T30-T0). The final result was expressed as the difference between the two scores, delta over baseline (DOB). A cutoff 3.5 DOB was used in accordance with the manufacturer's specifications. An increase above 3.5 DOB was considered positive for the presence of *H. pylori* infection.

### 2.3. Data Extraction

Demographic data and C13-UBT results were extracted from the central computerized CHS database. CHS is the largest health maintenance organization in Israel and the second largest health maintenance organization in the world, with more than 3.8 million enrollees. Data were retrieved and stored following the approval of the Institutional Review Board at Rabin Medical Center and according to the principles of the Declaration of Helsinki and Good Clinical Practice.

### 2.4. Statistical Analysis

All analyses were performed using SPSS version 24.0 statistical analysis software (IBM Inc., Chicago, IL, USA). The distributions of continuous variables were assessed for normality using the Kolmogorov-Smirnov test (cutoff at *p* < 0.01) and are described as means ± standard deviations (SD). Nominal variables were compared by using the chi-square test. Receiver operator curve (ROC) was plotted and analyzed with the Youden index. A multivariate forward logistic regression model was used in the statistical analysis to estimate odds ratios (OR) and 95% confidence interval. All tests were two-sided and considered significant at *p* < 0.05.

## 3. Results

A total of 94,590 subjects (36.1% male, age 28.5 ± 6.0 years) who underwent a first-time C13-UBT during the study period were included. C13-UBT was positive in 48,509 (51.3%) subjects. A confirmatory posttreatment C13-UBT was performed in 18,375 (37.8%), and eradication was successful in 12,018 (65.4%).

Among the 18,375 *H. pylori*-positive subjects who underwent a second C13-UBT, the mean initial C13-UBT recording was 20.6 ± 16.2 DOB among subjects with successful eradication and 19.5 ± 13.1 DOB in subjects with treatment failure (OR, 1.01; 95% CI 1.00-1.01, *p* < 0.01) (Table 1). ROC analysis determined that a cutoff of 14.9 DOB could predict treatment success with 45.2% sensitivity and 58.5% specificity.

**Figure 1:**
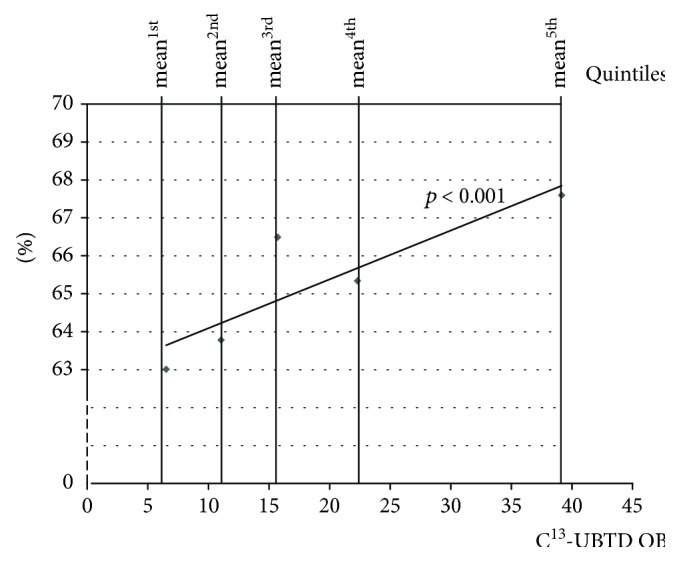
*H. pylori* eradication success according to the magnitude of the ^13^C-urea breath test by quintile.

Among the patients in the upper quintile of C13-UBT measurement, eradication was achieved in 67.6%, compared to 62.6% in the lower quintile (OR, 1.22; 95% CI 1.11-1.35, *p* < 0.01) (Figure 1). The results were similar when using a multivariate logistic regression model adjusted for age and sex (OR, 1.28; 95% CI 1.15-1.42; *p* < 0.01) (Table 2).

**Figure 2:**
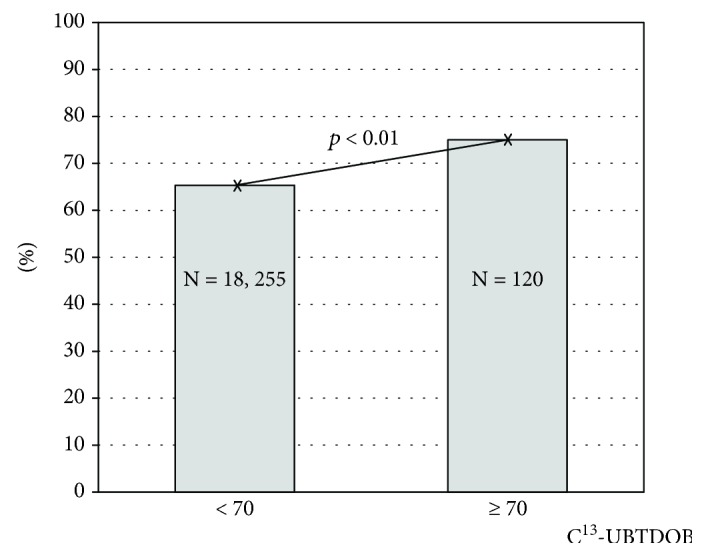
*H. pylori* eradication success in subjects with ^13^C-urea breath test measurement ≥ 70 DOB.

Subjects in the top 1 percentile with C13-UBT ≥ 70 DOB achieved eradication in 75.0%, compared to 65.3% among subjects with C13-UBT < 70 DOB (OR, 1.59; 95% CI 1.05-2.41, *p* < 0.01) (Figure 2). The results were similar when using a multivariate logistic regression model adjusted for age and sex (OR, 1.62; 95% CI 1.07-2.45, *p* < 0.01) (Table 2).

## 4. Discussion

We found that among subjects undergoing C13-UBT, as the DOB magnitude increases, the likelihood of successful eradication of *H. pylori* increases as well.

The magnitude of the DOB value is directly related to *H. pylori* urease activity. Therefore, it is unsurprising that DOB magnitude has been correlated with the density of *H. pylori* as measured by PCR, as well as by the grade of chronic active inflammation, as evidenced by infiltration of lymphocytes and neutrophils in the gastric mucosa [16–25, 32]. DOB magnitude has also been correlated with the frequency and intensity of dyspepsia [33]. Gastric mucosal atrophy, on the other hand, is associated with reduced bacterial density [34, 35]. It follows that subjects with a greater degree of gastric mucosal atrophy have lower DOB magnitude [23, 32]. A low PGI/II ratio, indicating gastric atrophy, has also been correlated with lower DOB magnitude [32]. It should be noted, however, that others did not find an association between bacterial density and DOB magnitude, although these studies did not include an assessment of chronic active inflammation [36, 37].

A previous study has examined the relationship between DOB magnitude and treatment success. In contrast to our findings, Gisbert et al. found no correlation between DOB magnitude and the likelihood of successful eradication, among 600 subjects [38]. Previous studies have examined the association between inflammation score, as measured by the revised Sydney System, and the likelihood of successful treatment [39]. Three studies have found that higher degrees of chronic active inflammation are associated with treatment success [27–29]. An additional study found that a high degree of mucosal atrophy decreases the likelihood of treatment success [30]. These studies did not include a molecular assessment of bacterial load or quantitative C13-UBT. However, if we consider the evidence that DOB magnitude is directly proportional to inflammatory cell infiltrate and inversely proportional to atrophy, as discussed, then these studies may be consistent with our findings, which suggest that DOB magnitude is proportional to treatment success. Taken together, it seems that subjects with a more severe inflammatory infiltrate are more likely to have a high bacterial load, a higher DOB, and greater treatment success. Subjects with mucosal atrophy and a lower bacterial load are more likely to have lower DOB magnitude and more treatment failure. Clearly, there is abundant overlap between these two scenarios, and while it may be a valid observation in a population setting, there is little relevance with respect to an individual patient.

There are several possible mechanisms why a high DOB value or inflammation score is associated with treatment success. Increased chronic active inflammation might trigger a favorable host response that facilitates eradication of the organism with appropriate treatment. Labenz et al. found that patients with successful eradication had a higher gastric pH compared with patients with treatment failure. A higher pH may augment the effect of amoxicillin by lowering the minimal inhibitory concentration, increase drug stability in the gastric lumen, and increase luminal concentration by slowing gastric emptying [40–42]. Nevertheless, the relationship between DOB magnitude and inflammation score is not completely clear, and these mechanisms might only explain why inflammation, but not DOB, is related to treatment success.

A limitation of our study is the lack of histopathology data to correlate our findings. Another limitation is the likely presence of multiple confounders which cannot be accounted for. Firstly, there are multiple factors which determine DOB magnitude, besides bacterial density, which may account for the observed difference in treatment success. These include patient factors, bacterial factors, and test/laboratory factors [6–15]. Secondly, there are confounding factors which may account for differences in treatment success. These include drug compliance, antibiotic resistance, antibiotic regimen, drug-drug interactions, CYP2C19 polymorphisms, and smoking. Nevertheless, given the large sample size, it is unlikely that controlling for these factors would significantly alter the results. Another limitation is the relatively low eradication rate of 65.4%. Previous data suggest that over 90% of subjects received clarithromycin-based triple therapy [43], despite the fact that primary resistance of *H. pylori* to clarithromycin in our region is >20% [44, 45]. According to the current treatment guidelines, clarithromycin-based triple therapy is not recommended in regions where the primary clarithromycin resistance exceeds 15% [1, 46]. Nevertheless, clarithromycin-based triple therapy remains by far the most common treatment protocol utilized [43]. If subjects had received more efficacious treatment regimens with a higher eradication rate, it is possible that the DOB magnitude would no longer be significantly associated with treatment success.

For the purpose of this study, we assumed that antibiotic treatment was administered following a positive C13-UBT and prior to a subsequent C13-UBT. Some subjects, however, may not have received treatment or may have received more than one treatment course prior to repeating C13-UBT. We have no data on the various treatment protocols used. Although we excluded subjects above the age of 45 years, some subjects may have undergone endoscopy-based tests for *H. pylori* diagnosis and may have received treatment prior to the index test. Finally, our cohort is limited to 18-45-year-olds. We excluded subjects over the age of 45 since these patients were more likely to have undergone an upper gastrointestinal endoscopy as their initial test for dyspepsia. Otherwise, patients with a positive C13-UBT following a positive endoscopy-based test and subsequent treatment would have been inaccurately categorized as treatment naïve. Since we could not reliably identify patients who had previously undergone an endoscopy-based test for *H. pylori*, we chose to exclude patients > 45 years old. Our results may not be applicable to older subjects with more longstanding infection, higher rates of gastric atrophy, and perhaps better treatment compliance. The strength of our study lies in the large cohort, the rigorous exclusion criteria, and the quality of the database used.

In conclusion, we found that higher DOB magnitude is associated with a greater degree of successful eradication of *H. pylori*. Further studies which incorporate histopathological and clinical variables are needed to verify and elucidate the physiological basis for our findings.

## Figures and Tables

**Table 1:** Patient characteristics.

	Treatment success	Treatment failure	*p*
*N* (%)	12,018 (65.4)	6357 (34.6)	
C13-UBT DOB, mean (SD)	20.6 (16.2)	19.5 (13.1)	<0.01
Sex, male, *N* (%)	4264 (35.5)	2121 (33.4)	<0.01
Age, years, mean (SD)	28.9 (5.9)	28.4 (5.9)	<0.01

Abbreviations: C13-UBT: ^13^C-urea breath test; DOB: delta over baseline.

**Table 2:** Factors associated with successful eradication of *H. pylori* (multivariate analysis).

	OR	95% CI	*p*
C13-UBT ≥ 70 DOB	1.62	1.07-2.45	<0.01
Upper quintile DOB	1.28	1.15-1.42	<0.01
Male sex	1.13	1.06-1.21	<0.01
Age	1.02	1.01-1.02	<0.01

Abbreviations: C13-UBT: ^13^C-urea breath test; DOB: delta over baseline.

## Data Availability

The raw data used to support the findings of this study are available from the corresponding author upon request.
